# Prevalence and predictors of malignancy in Bethesda III and IV thyroid nodules undergoing surgery

**DOI:** 10.3389/fendo.2026.1859531

**Published:** 2026-08-17

**Authors:** Anna Krzentowska, Aleksander Józef Konturek, Filip Gołkowski, Anna Merklinger-Gruchała, Marcin Barczyński

**Affiliations:** 1 Department of Endocrinology and Internal Medicine, Medical College, Andrzej Frycz Modrzewski Krakow University, Kraków, Poland; 2 Department of Endocrine Surgery, Faculty of Medicine, Jagiellonian University Medical College, Kraków, Poland; 3 Department of Bioinformatics and Public Health, Medical College, Andrzej Frycz Modrzewski Krakow University, Kraków, Poland

**Keywords:** Bethesda III, Bethesda IV, malignancy, predictors, thyroid cancer

## Abstract

**Background:**

Indeterminate thyroid cytology remains a major diagnostic challenge. Thyroid nodules classified as Bethesda category (BC) III and IV carry an intermediate risk of malignancy, and their management often involves a choice between active surveillance and diagnostic surgery. This study aimed to assess the prevalence of benign, low-risk, and malignant lesions in surgically treated patients with these nodules, and to identify factors associated with malignancy.

**Methods:**

We retrospectively analyzed 618 patients with BC III or IV thyroid nodules, selected from 2,239 patients who underwent thyroid surgery between October 2022 and December 2025 at a Tertiary Referral Endocrine Surgery Centre in Krakow. Histopathology followed the 2022 WHO Classification of Thyroid Tumors.

**Results:**

The distribution of tumor types differed significantly in both BCs III (n=365) and IV (n=253) nodules (both p<0.001), with benign lesions predominating (62.5% in BC III and 55.7% in BC IV), followed by malignant (30.4% and 32.8%) and low-risk lesions (7.1% and 11.5%), respectively. Younger age was associated with a higher risk of malignancy in both BC III (p=0.03) and BC IV (p=0.02). Sex was associated with lesion distribution in BC III (p = 0.016) but not BC IV (p = 0.06), and was not an independent predictor of malignancy. Tumor size ≥1 cm was associated with malignancy in BC IV (p=0.005), but not in BC III (p=0.35). Although age alone showed limited discriminative ability, multivariable analysis identified age <50 years as the only independent predictor of malignancy (adjusted OR 1.74; 95% CI 1.23–2.47; p=0.002) and lymph node metastasis (adjusted OR 4.56; 95% CI 1.19–17.53; p=0.03), while sex, chronic lymphocytic thyroiditis (CLT), and BC were not significant.

**Conclusions:**

In this surgical cohort of BC III and IV thyroid nodules, approximately one-third of lesions were malignant. Younger age was independently associated with malignancy, but its value as an isolated predictor was limited, and the overall discriminative performance of the multivariable model was modest. These findings may support individualized preoperative risk stratification; however, given the retrospective design and the selection of patients for surgery, they do not justify firm conclusions regarding the optimal extent of surgery.

## 1 Introduction

The Bethesda System for Reporting Thyroid Cytopathology (TBSRTC) established a standardized, category-based reporting system for thyroid fine-needle aspiration biopsy (FNAB) specimens (1). Thyroid ultrasound (US) and FNAB are the primary diagnostic tools for thyroid nodules (2, 3). The Bethesda classification of thyroid FNA cytology is used for diagnostic classification and therapeutic recommendations. Currently, there is a division into six BCs: I – nondiagnostic; II – benign; III – atypia of undetermined significance (AUS) or follicular lesion of undetermined significance (FLUS); IV –follicular neoplasm; V – suspicious for malignancy; and VI – malignant. According to the 2022 World Health Organization (WHO) Classification of Thyroid Tumors, thyroid neoplasms are categorized as benign, low-risk, or malignant (4). Malignant tumors include papillary thyroid carcinoma (PTC), follicular thyroid carcinoma (FTC), oncocytic thyroid carcinoma (OTC), medullary thyroid carcinoma (MTC), and anaplastic thyroid carcinoma (ATC). Low-risk thyroid neoplasms (LRTN) comprise non-invasive follicular thyroid neoplasm with papillary-like nuclear features (NIFTP), follicular tumor of uncertain malignant potential (FT-UMP), well-differentiated tumor of uncertain malignant potential (WDT-UMP), and hyalinizing trabecular tumor (HTT) (5).

For nodules classified as BCs V and VI, surgery is the treatment of choice. In contrast, thyroid nodules classified as BCs III and IV remain a clinical challenge, particularly in determining whether to proceed with surgical management or to adopt active surveillance with ultrasound follow-up. Surgical treatment, together with subsequent histopathological evaluation, provides a definitive diagnosis. However, patients typically require lifelong levothyroxine therapy due to hypothyroidism. Therefore, for nodules classified as BCs III and IV, it is crucial to assess whether surgical intervention is absolutely necessary or if alternative management strategies, such as active surveillance or repeat FNAB, can be considered. Clinical decision-making should take into account the estimated risk of malignancy, sonographic characteristics, patient comorbidities and preferences, and the long-term implications of thyroid hormone replacement therapy. Numerous studies have evaluated the risk of malignancy in these categories (6–12). Reported malignancy rates vary, with estimates of 29.6% and 47.1% (6), 25% for BC III and 27.6% for BC IV (7), 26.3% and 26.6% (8), 38% and 58% (9), and 18.2% and 33.3% (12), respectively. While molecular testing can provide additional information to estimate malignancy risk, these methods are not widely available in routine clinical practice in Europe. Consequently, the primary tools for evaluation remain US and FNAB performed under US guidance. Several studies have examined the ultrasonographic characteristics of nodules classified as BCs III and IV, demonstrating that features such as hypoechogenicity and the presence of microcalcifications are associated with an increased risk of malignancy (13, 14). Other sonographic features, including the size of the dominant nodule, irregular margins, and a taller-than-wide shape, have also been reported to influence malignancy risk (13).

Although the prevalence and predictors of malignancy in thyroid nodules classified as BCs III and IV have been extensively investigated, previous studies have primarily focused on the assessment of malignancy risk, with histopathological outcomes most commonly reported using a dichotomous classification of benign versus malignant lesions (6–12). LRTN have not been routinely evaluated as a separate category of histopathological outcomes in studies assessing Bethesda III and IV nodules. The 5th edition of the WHO Classification of Thyroid Tumors published in 2022 further refined the classification of thyroid neoplasms by recognizing LRTN, including NIFTP, FT-UMP, WDT-UMP, and HTT, as a distinct group with clinical and biological characteristics different from conventional malignant thyroid tumors (5). Therefore, histopathological outcomes of Bethesda III and IV nodules require further characterization using a classification that incorporates this distinct diagnostic category. Data regarding the prevalence of LRTN and factors associated with their occurrence in large cohorts of surgically treated Bethesda III and IV nodules remain limited.

In light of the evolving classification of thyroid neoplasms and the remaining gaps in available evidence, we aimed to characterize the histopathological outcomes of thyroid nodules classified as BCs III and IV by evaluating the prevalence of benign lesions, LRTN, and malignant tumors in a cohort of surgically treated patients.

The objectives of this study were as follows:

Primary objective: to determine the prevalence of benign lesions, LRTN, and malignant tumors among nodules classified as BCs III and IV.
Secondary objective: to identify factors associated with an increased risk of malignancy in nodules classified as BCs III and IV and to identify factors influencing the risk of lymph node metastases among patients with histopathologically (HP) confirmed thyroid cancer (TC).

## 2 Materials and methods

### 2.1 Study group

**Figure 1:**
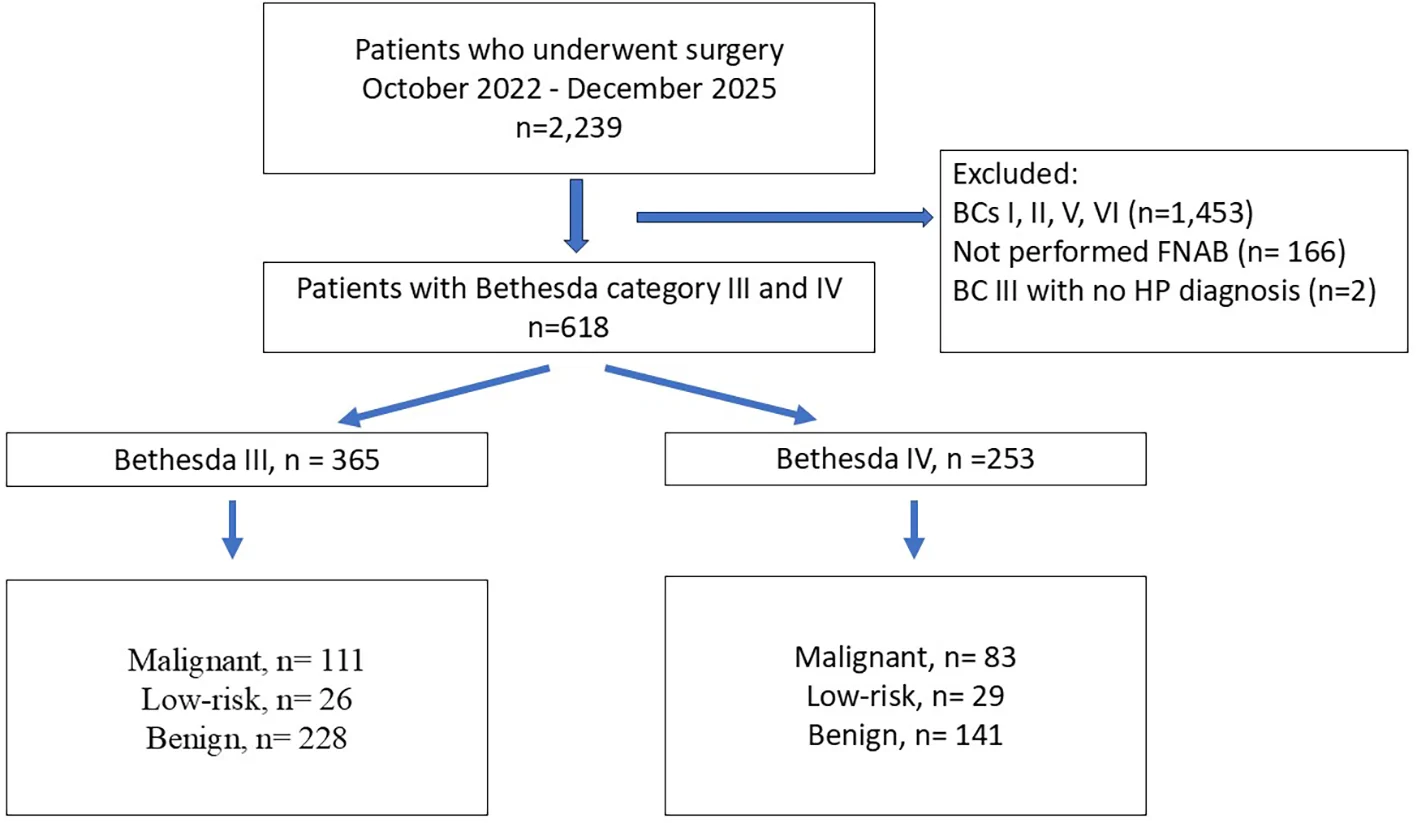
Flowchart of the inclusion or exclusion procedure.

We conducted a retrospective analysis of 2,239 patients who underwent surgery at the Department of Endocrine Surgery, Jagiellonian University Medical College, based at the University Hospital in Krakow, between October 2022 and December 2025. Patients were referred for surgery due to nodular goiter (NG), hyperthyroidism associated with Graves’ disease (GD) or toxic multinodular goiter (TMNG) and confirmed or suspected thyroid cancer (TC) based on FNAB. Patients with BCs I, II, V, and VI were excluded from the analysis. Consequently, only patients with BCs III and IV were included in the study. Patient data were anonymized and collected in the international EUROCRINE Registry database (www.eurocrine.eu; last accessed on March 29, 2026). The study was approved by the Bioethics Committee of Andrzej Frycz Modrzewski Krakow University (approval no. KB/UAFM/8/O/2025, issued on 23 January 2025). A visual representation of the patient selection process is presented in **Figure 1**.

### 2.2 Diagnosis and evaluation

All patients underwent thyroid US and FNAB of suspicious thyroid nodules prior to surgery. Patients were referred for surgical treatment from various medical centers; therefore, not all nodules had been assessed according to the EU-TIRADS classification. Due to missing data, EU-TIRADS assessment was not included in the analysis. FNAB results were reported according to the BSRTC. Patients underwent either total thyroidectomy or thyroid lobectomy. The extent of lymph node dissection was determined based on the preoperative FNAB result. Postoperative specimens were evaluated HP, which served as the reference standard for confirming or excluding thyroid malignancy. Histopathological assessment was performed according to the 2022 WHO Classification of Thyroid Tumors (2022). Low-risk thyroid neoplasms (LRTN) were defined according to the 2022 WHO Classification of Thyroid Tumors as a distinct category of thyroid neoplasms characterized by indolent biological behavior and a very low risk of adverse outcomes compared with malignant thyroid tumors. LRTN included NIFTP, FT-UMP, WDT-UMP, and HTT. For the purpose of this study, LRTN were analyzed as a separate histopathological outcome category, distinct from both benign lesions and malignant tumors. Therefore, final histopathological diagnoses were classified into three groups: benign lesions, LRTN, and malignant tumors. In patients with BCs III and IV, the frequencies of malignant tumors, LRTN, and benign lesions were analyzed. In both groups, the following variables were analyzed: age, sex, HP diagnosis, type of malignant thyroid tumor, CLT confirmed by HP examination, and the extent of thyroid and lymph node surgery. If malignancy was confirmed, tumor staging was performed according to the AJCC/UICC TNM Classification of Malignant Tumors, 8th Edition (2017).

### 2.3 Statistical analysis

Continuous variables were expressed as mean ± SD and median and quartiles (Q1, Q3) and compared using the Kruskal–Wallis test. The categorical variables were expressed as numbers (percentages). The χ2 test with *post-hoc* test and calculated standardized residuals (SR) or Fisher’s exact test or two-proportion test were used to compare groups. The observed distribution of diagnoses (Benign, Low-risk, Malignant) among BCs nodules was assessed using Pearson χ² goodness of fit test. Differences in the distribution of histological cancer subtypes between BCs III and IV were assessed using the Fisher–Freeman–Halton exact test.

To identify predictors of malignancy in thyroid nodules, simple and multivariate logistic regression analysis was performed. Variables demonstrating associations in univariate analyses, including sex, CLT confirmed by HP examination and cytological category, together with age, which was included because of its established clinical relevance, were entered into the multivariable model.

Furthermore, simple and multivariate logistic regression analyses were performed to identify independent predictors of lymph node metastasis among patients with HP confirmed TC, including age, sex, CLT, tumor size, and BCs in the model. Model fit was evaluated using the Hosmer–Lemeshow test, discrimination by using the AUC, and explained variance by using Cox–Snell and Nagelkerke R².

In all logistic regression models, age was included as a categorical variable; the cut-off value of 50 years was determined based on ROC analysis, where age was treated as a continuous variable. Although the ROC analysis showed limited discriminative ability of age as an isolated predictor (AUC = 0.55), the threshold corresponding to the highest Youden’s J statistic was used for dichotomization, and age was retained in the multivariable models because of its established clinical relevance.

Results with p-values < 0.05 were considered statistically significant. All statistical analyses were performed using Statistica 13 (StatSoft Inc.) and jamovi software version 2.6.26.

## 3 Results

### 3.1 Baseline characteristics

A total of 2,239 individuals (n = 428 males, n = 1811 females, with a median age of 56 years) who had undergone unilateral lobectomy or total thyroidectomy for thyroid nodules between October 2022 and December 2025. The FNAC results were as follows: 86 (3.8%) BC I; 817 (36.5%) BC II; 365 (16.4%) BC III; 253 (11.3%) BC IV; 299 (13.4%) BC V; 251 (11.2%) BC VI; and 166 (7.4%) not performed FNAB. HP revealed malignant lesions in 824 of the 2,239 patients included in the study cohort. The most common malignancy was PTC (n = 691), followed by FTC (n = 62), MTC (n = 36), OTC (n = 28), ATC (n = 3), and lymphoma (n = 4).

Since BCs III and IV remain a clinical challenge, our further analysis focused specifically on these two subgroups in order to assess the prevalence of malignancy. Of the 618 patients with BCs III and IV, 514 (83.1%) were females and 104 (16.9%) were males with a median age of 55.0 years (IQR = 23.0 years). Based on HP, among patients with BCs III and IV nodules, 369 (59.5%) lesions were benign, 194 (31.2%) were malignant, and 55 (8.8%) were classified as lesions with low risk. Statistically significant differences between BCs III and IV were observed in terms of the maximum malignant tumor size (p = 0.015), malignant tumor size subcategories (<1 cm vs. ≥1 cm; p = 0.007), and the weight of the thyroid gland removed following total thyroidectomy (p = 0.001). The tumor size was determined only for malignant tumors based on histopathological data (**Table 1**).

**Table 1:** Characteristics of patients undergoing surgery for BCs III and IV thyroid nodules.

Characteristic	Cytology III (n=365)	Cytology IV (n=253)	P-value
Age (years),			0.21
mean (SD)	53.7 (13.7);	55.2 (15.7);	
median (Q1-Q3)	55.0 (43.0–65.0)	56.0 (42.0–68.0)	
Sex			0.60
Female	306 (83.8%)	208 (82.2%)	
Male	59 (16.2%)	45 (17.8%)	
Malignant largest tumor size (mm),			0.02
mean (SD)	13.0 (12.1);	17.4 (14.2);	
median (Q1-Q3) †	8.0 (5.0–17.0)	14.0 (7.0–24.0)	
N	107	81	
Malignant tumor size category, n (%) ^†^			
<1 cm	58 (52.3%)	28 (34.6%)	0.01
≥1 cm	49 (44.1%)	53 (65.4%)	
	47 (12.8%)	36 (14.2%)	0.61
CLT, n (%) ^‡^			0.10
Malignant	111 (30.4%)	83 (32.8%)	
Low-risk	26 (7.1%)	29 (11.5%)	
Benign	228 (62.5%)	141 (55.7%)	
Malignant lymph node status, n (%),			0.60
pN0	77 (78.6%)	58 (74.4%)	
pN1a	6 (6.1%)	8 (10.2%)	
pNx	15 (15.3%)	12 (15.4%)	
Total thyroid gland weight (g), mean (SD) / median (Q1-Q3)			
After total thyroidectomy	36.5 (35.8) 26.0 (17.0–44.0)	26.7 (19.5); 21.0 (14.0–34.0)	<0.01
N	276	195	
After lobectomy	17.3 (15.1); 11.0 (8.0–22.0)	14.4 (19.7); 9.0 (7.0–13.0)	0.13
N	53	37	
Type of thyroid surgery, n (%)	367	253	
Total thyroidectomy	303 (83.0%)	214 (84.6%)	
Unilateral lobectomy of thyroid gland	62 (17.0%)	39 (15.4%)	
Type of lymph node surgery, n (%)			
None	35 (9.6%)	11 (4.4%)	
Unilateral central lymph node dissection	132(36.2%)	101 (39.9%)	
Bilateral central lymph node dissection	180 (49.3%)	129 (51.0%)	
Bilateral lateral lymph node dissection	0 (0.0%)	1 (0.4%)	
Central lymph node dissection (PJD41) and one-sided lat. Lymph node dissection	18 (4.9%)	10 (4.0%)	
One-sided lateral lymph node dissection	0 (0.0%)	1 (0.4%)	

† Tumor size available only for cases with histopathological measurement.

‡ Among patients with histological secondary diagnosis recorded.

### 3.2 Univariate analysis of thyroid nodules in patients with BCs III and IV

#### 3.2.1 Univariate analysis of focal lesion types in patients with BC III nodules (n=365)

Subsequently, histopathological results were analyzed exclusively in patients with BC III nodules in order to determine the prevalence of malignancy within this category. Statistically significant differences were observed in the frequency of benign, malignant, and low-risk lesions (p < 0.001), age (p = 0.03), as well as in sex distribution (p = 0.016). The results are presented in **Table 2**.

**Table 2:** Analysis of focal lesion types (benign, low-risk, and malignant) among BC III nodules (n = 365).

Characteristic	Benign	Low-risk	Malignant	P-value
No. of patients, n (%)	228 (62.5)	26 (7.1)	111 (30.4)	<0.001^$^
Age, years				0.03*
Mean (SD)	55.2 (13.2)	51.7 (14.4)	51.0 (14.2)	
Median (Q1-Q3)	58.5 (44.5–65.5)	51.0 (36.0–66.0)	48.0 (41.0–63.0)	
Sex, n (%)				0.016†
Female	200 (65.4)	18 (5.9)	88 (28.8)	
Male	28 (47.5)	8 (13.6)	23 (39.0)	
Malignant largest tumor size, mm				
Mean (SD)	—	—	13.0 (12.1)	
Median (Q1-Q3)	—	—	8.0 (5.0–17.0)	
Malignant tumor size category, n (%)				0.35‡
<1 cm	—	—	58 (52.3)	
≥1 cm	—	—	49 (44.1)	
Missing			4	
CLT, n (%)	25 (53.2)	2 (4.3)	20 (42.6)	<0.001*^$^*
Histopathology, n (%)	NG – 141 (62.7) FA – 59 (25.9) OA – 11 (4.8) CLT – 15 (6.6)	FT-UMP – 11 (42.3) NIFTP – 8 (30.8) WDT-UMP – 7 (26.9)	PTC – 81 (73.0) FTC – 18 (16.2) MTC – 7 (6.3) OTC – 4 (3.6) Lymphoma – 1 (0.9)	

$ Pearson χ² goodness of fit; * Kruskal–Wallis test; † Pearson χ² test; ‡ Two-proportion test for the malignant group, excluding missing values.

NG, Nodular goiter; FA, Follicular adenoma; OA, Hürtle cell adenoma; CLT, chronic lymphocytic thyroiditis; FT-UMP, Follicular tumor with uncertain malignant potential; NIFTP, Non-invasive follicular thyroid neoplasm with papillary-like nuclear features; WDT-UMP, Well differentiated tumor of uncertain malignant potential; PTC, papillary thyroid cancer; FTC, follicular thyroid cancer; MTC, medullary thyroid cancer; OTC, oncocytic thyroid cancer.

Among the 365 lesions, 62.5% were benign, 7.1% were low-risk neoplasms, and 30.4% were malignant. The distribution of lesion types differed significantly from uniformity (χ²(2) = 169.1, p < 0.001). Pairwise comparisons of proportions with Bonferroni correction showed that benign lesions occurred significantly more frequently than malignant lesions (p < 0.001) and low-risk lesions (p < 0.001). Additionally, malignant lesions were observed significantly more often than low-risk lesions (p < 0.001).

Age was significantly associated with lesion type (Kruskal–Wallis test: H(2, N = 365) = 7.4, p = 0.03), and *post-hoc* multiple comparisons revealed that patients with malignant lesions were significantly younger than those with benign lesions (p = 0.03).

A significant association between sex and lesion type was also observed (Pearson χ²(2) = 8.33, p = 0.016). *Post-hoc* analysis of standardized residuals showed that benign lesions were more common in women than in men (SR = 2.60), while low-risk lesions were more common in men (SR = 2.10). No significant gender differences were found in the frequency of malignant lesions (SR = 1.56).

The proportion of lesions ≥1 cm among malignant nodules did not differ significantly (p = 0.35).

The distribution of tumor types in patients with CLT confirmed in HP differed significantly from the uniform distribution (χ²(2) = 18.68, p < 0.001). *Post hoc* pairwise comparisons with Bonferroni correction (α = 0.0167) showed that both malignant and benign lesions were significantly more common than low-risk lesions (both p < 0.001), whereas no significant difference was observed between malignant and benign lesions (p = 0.551).

#### 3.2.2 Univariate analysis of nodules in patients with BC IV (n=253)

A similar analysis was conducted among patients with BC IV nodules to assess lesion types based on HP examination. Statistically significant differences were observed in the frequency of benign, malignant, and low-risk lesions, as well as in age and tumor size subcategories (<1 cm and ≥1 cm). Tumor size was determined based on histopathology only for malignant lesions; therefore, data on the dimensions of benign and low-risk lesions were not available (**Table 3**).

**Table 3:** Analysis of focal lesion types (benign, low-risk, and malignant) among BC IV nodules (n = 253).

Characteristic	Benign	Low-risk	Malignant	P-value
No. of patients, n (%)	141 (55.7)	29 (11.5)	83 (32.8)	<0.001$
Age, years				0.02*
Mean (SD)	56.8 (16.2)	47.8 (13.3)	54.9 (14.9)	
Median (Q1–Q3)	59.0 (44.0–70.0)	48.0 (39.0–56.0)	55.0 (42.0–68.0)	
Sex, n (%)				0.058†
female/male	122 (58.7)/19 (42.2)	20 (9.6)/9 (20.0)	66 (31.7)/17 (37.8)	
Malignant largest tumor size, mm				
Mean (SD)	—	—	17.4 (14.2)	
median (Q1–Q3)	—	—	14.0 (7.0–24.0)	
Malignant tumor size category, n (%)				0.005‡
<1 cm	—	—	28 (34.6)	
≥1 cm	—	—	53 (65.4)	
Missing			2	
CLT, n (%)	22 (61.1)	3 (8.3)	11 (30.6)	<0.001*$*
Histopathology, n (%)	NG – 49 (35.4) FA – 40 (28.4) OA – 45 (31.9) CLT – 4 (2.8) Other diagnosis – 2 1.4)	FT-UMP – 10 (34.5) NIFTP – 13 (44.8) WDT-UMP – 6 (20.7)	PTC – 42 (50.6) FTC – 23 (27.7) OTC – 17 (20.5) MTC – 1 (1.2)	0.73*†*

$ Pearson χ² goodness of fit; * Kruskal–Wallis test; † Pearson χ² test; ‡ Two-proportion test for the malignant group, excluding missing values.

NG, Nodular goiter; FA, Follicular adenoma; OA, Hürtle cell adenoma; CLT, chronic lymphocytic thyroiditis; FT-UMP, Follicular tumor with uncertain malignant potential; NIFTP, Non-invasive follicular thyroid neoplasm with papillary-like nuclear features; WDT-UMP, Well differentiated tumor of uncertain malignant potential; PTC, papillary thyroid cancer; FTC, follicular thyroid cancer; MTC, medullary thyroid cancer; OTC, oncocytic thyroid cancer.

Among the n=253 nodules, 55.7% were benign, 11.5% were classified as low-risk, and 32.8% were malignant. The distribution of tumor types differed significantly from a uniform distribution (p<0.001). Pairwise proportion comparisons with Bonferroni correction showed that benign nodules occurred significantly more frequently than malignant nodules (p<0.001) and intermediate-risk lesions (p<0.001). In addition, malignant nodules were observed significantly more often than intermediate-risk lesions (p<0.001).

Age was significantly associated with tumor type (Kruskal–Wallis test: H(2, N = 253)=8.2, p=0.017), and *post-hoc* multiple comparisons indicated that patients with benign nodules were significantly older than those with low-risk lesions (p=0.014). No significant age differences were observed between patients with malignant versus benign nodules (p=0.953) or between malignant and low-risk nodules (p>0.123).

Sex was not significantly associated with tumor type (Pearson χ²(2)=5.7, p=0.058), but trends were noted: benign lesions were more frequently observed in women, whereas the proportion of malignant and benign nodules was similar in men.

The proportion of nodules ≥1 cm among malignant nodules differed significantly (Pearson χ²(1)=7.7, p=0.005), with nodules ≥1 cm occurring more frequently than those <1 cm (65.4% vs. 34.6%).

The distribution of tumor types in patients with CLT differed significantly from a uniform distribution (χ²(2) = 15.2, p < 0.001). *Post-hoc* pairwise comparisons with Bonferroni correction (α = 0.0167) showed that benign lesions were significantly more frequent than low-risk lesions (p < 0.001), while no statistically significant differences were observed between malignant and low-risk lesions (p = 0.057) or between malignant and benign lesions (p = 0.080), although a trend toward a higher frequency of benign compared to malignant lesions was noted.

### 3.3 ROC analysis for determination of optimal age cutoff

Receiver operating characteristic (ROC) curve analysis with the Youden index was performed to determine the optimal age cut-off. The analysis identified 50 years as the optimal threshold (AUC = 0.55; 95% CI: 0.50–0.60), corresponding to a sensitivity of 53% and specificity of 63% (Youden index = 0.15). Although the AUC indicates limited discriminative ability of age as an isolated predictor of malignancy, the 50-year threshold was used to dichotomize age for regression analyses. The ROC curve is presented in the **Supplementary Material** (**Supplementary Figure 1**).

### 3.4 Subtypes of PTC and FTC in thyroid nodules of BCs III and IV

In this section, we analyzed the distribution of PTC and FTC subtypes among nodules classified as BCs III and IV. This assessment aimed to identify the relative frequencies of each histological subtype within these cytological risk groups (**Table 4**). The distribution of histological cancer subtypes differed significantly between BCs III and IV (Fisher–Freeman–Halton exact test, p=0.001). In nodules classified as BC IV, the follicular variant of PTC predominated (88.5%). In contrast, nodules classified as BC III showed a more heterogeneous histological pattern, with the follicular variant of PTC being the most common subtype (50.0%), followed by the FTC minimal invasive subtype (26.1%). FTC subtypes were observed exclusively in the Bethesda III category (39.2%).

**Table 4:** PTC and FTC subtypes among tumors with BC III and IV.

Subtype of malignant tumor	Bethesda III (n=46)	Bethesda IV (n=26)	P-value*
Follicular subtype of PTC	23 (50.0%)	23 (88.5%)	0.001
Encapsulated subtype of PTC	0	1 (3.9%)	
Other unusual subtype of PTC:	5 (10.9%)	2 (7.7%)	
Tall-cell PTC – 2 cases			
Warthin-like PTC – 1 case			
Diffuse sclerosing PTC – 1 case			
Infiltrative follicular PTC – 2 cases			
Oncocytic – 1 case			
FTC minimal invasive	12 (26.1%)	0	
FTC encapsulated angioinvasive	5 (10.9%)	0	
FTC widely invasive	1 (2.2%)	0	

*Fisher–Freeman–Halton exact test.

### 3.5 Multivariate analysis of risk factors for malignant lesions

To identify independent predictors of malignancy in thyroid nodules, a multivariate logistic regression analysis was performed. Variables showing potential associations in univariate analyses, including sex, CLT and cytological category, together with patient age, which was retained in the model because of its established clinical relevance despite its limited discriminative performance in the univariate ROC analysis, were included in the model. Tumor size was not considered due to the lack of available measurements for benign and low-risk lesions (**Table 5**).

**Table 5:** Univariate (crude) and multivariate (adjusted) logistic regression models assessing predictors of risk of malignancy.

Predictors	Crude		Adjusted	
	OR (95% CI)	P-value	OR (95% CI)	P-value
Sex (male vs female)	1.46 (0.94–2.26)	0.090	1.44 (0.92–2.26)	0.109
Bethesda category (IV vs III)	1.12 (0.79–1.58)	0.528	1.13 (0.80–1.61)	0.485
Age (<50 vs ≥50)	1.79 (1.27–2.53)	0.001	1.74 (1.23–2.47)	0.002
CLT (yes vs no)	1.36 (0.84–2.20)	0.210	1.37 (0.84–2.24)	0.214

Odds ratios (OR) and 95% confidence intervals (CI) are presented.

Adjusted model fit statistics: Hosmer–Lemeshow χ2 = 3.5, p = 0.62; AUC = 0.60 (SE = 0.02); Cox–Snell R2 = 0.02; Nagelkerke R2 = 0.03.

In the univariate analysis, men compared to women showed a trend towards higher risk of malignancy; however, this association did not reach statistical significance either before (OR 1.46; 95% CI: 0.94–2.26; p = 0.090) or after adjustment for age, CLT status and BC (adjusted OR 1.44; 95% CI: 0.92–2.26; p = 0.10); p = 0.148). Similarly, CLT was not significantly associated with malignancy risk in either univariate (OR 1.36; 95% CI: 0.84–2.20; p = 0.210) or multivariate analysis (adjusted OR = 1.37; 95% CI: 0.84–2.24). BC IV (vs III) was not a significant predictor of malignancy either before (OR 1.12; 95% CI: 0.79–1.58) or after adjustment (adjusted OR 1.14; 95% CI: 0.80–1.61; p = 0.467). In contrast, age <50 years (vs ≥50 years) was significantly associated with a higher risk of malignancy both in univariate analysis (OR 1.79; 95% CI: 1.27–2.53; p = 0.001) and after adjustment for other variables (adjusted OR 1.74; 95% CI: 1.23-2.47; p = 0.002), thus confirming its role as an independent predictor.

The model demonstrated good fit to the data (Hosmer–Lemeshow p=0.62); however, its discriminative ability was limited (AUC = 0.60), and the low pseudo-R² values (Cox–Snell R²=0.02; Nagelkerke R²=0.03) suggest that the included variables explain only a small proportion of the variability in malignancy risk.

### 3.6 Multivariate analysis of lymph node metastasis risk among malignant thyroid nodules

To evaluate factors influencing the presence of lymph node metastases among patients with HP-confirmed TC, a multivariate analysis was performed. The model included age, sex, tumor size, CLT status and BC to assess their independent effects on lymph node involvement (**Table 6**).

**Table 6:** Univariate (crude) and multivariate (adjusted) logistic regression models assessing predictors of risk of metastasis among malignant tumors.

Predictors	Crude		Adjusted	
	OR (95% CI)	P-value	OR (95% CI)	P-value
Sex (male vs female)	1.26 (0.33–4.48)	0.736	1.17 (0.28–4.85)	0.826
Bethesda category (IV vs III)	1.77 (0.58–5.38)	0.314	2.21 (0.68–7.23)	0.190
Largest tumor size (>=1 vs <1cm)	1.06 (0.35–3.20)	0.914	0.94 (0.28–3.08)	0.913
Age (<50 vs ≥50)	4.45 (1.19–16.67)	0.027	4.56 (1.19-17.53)	0.027
CLT status (yes vs no)	1.48 (0.38–5.76)	0.571	1.38 (0.33–5.86)	0.662

Odds ratios (OR) and 95% confidence intervals (CI) are presented.

Adjusted model fit statistics: Hosmer–Lemeshow χ2 = 6.3, p = 0.39; AUC = 0.71 (SE = 0.08); Cox–Snell R2 = 0.05; Nagelkerke R2 = 0.11.

Restricting the analysis to patients with TC, no significant association was observed between BC and the risk of lymph node metastases, either in unadjusted analysis (OR 1.77; 95% CI: 0.58–5.38; p=0.314) or after adjustment for additional factors (adjusted OR 2.21; 95% CI: 0.68–7.23; p=0.190), although a nonsignificant trend toward higher risk in BC IV versus III persisted. Sex (adjusted OR 1.17; 95% CI: 0.28–4.85; p=0.826) and malignant tumor size ≥1 cm (adjusted OR 0.94; 95% CI: 0.28–3.08; p=0.913) were also not associated with lymph node metastases. The only significant and independent predictor was age <50 years (vs. ≥50 years), which was associated with an approximately 4.5-fold increased risk of metastases both in univariate analysis (OR 4.45; 95% CI: 1.19–16.67; p = 0.027) and multivariate analysis (adjusted OR 4.56; 95% CI: 1.19–17.53; p=0.027), with minimal change in OR after adjustment, indicating the stability of the effect.

Overall, the analysis suggests that younger age is the primary factor associated with an increased risk of lymph node metastasis, whereas sex, BC, tumor size, and CLT do not independently predict metastatic spread in this cohort.

The adjusted model demonstrated good calibration (Hosmer–Lemeshow χ²=6.3; p=0.39) and moderate discriminative ability (AUC = 0.71; SE = 0.08), thus suggesting acceptable classification utility. However, the relatively low pseudo-R² values (Cox–Snell R²=0.05; Nagelkerke R²=0.11) indicate that the model explains only a limited proportion of the variability in metastatic risk, and the wide confidence intervals (particularly for age) reflected uncertainty in the estimates. The multivariable logistic regression model was fitted to 146 complete cases, including 14 patients with central lymph node metastasis (events) and 132 without metastasis (non-events). Five predictors were included in the model, yielding an events-per-variable (EPV) ratio of 2.8. This relatively low EPV suggests an increased risk of model overfitting and limited stability of the regression coefficients; therefore, the results should be interpreted with caution.

## Discussion

In this retrospective cohort of surgically treated patients with BCs III and IV thyroid nodules, malignancy was identified in approximately one-third of cases in both cytological categories. The observed rates were 30.4% for BC III and 32.8% for BC IV and are broadly consistent with previously published surgical series (**Table 7**).

**Table 7:** The rate of malignancy among Bethesda category III and IV in other studies.

References	Malignant in BC III (%)	Malignant in BC IV (%)
Zahid A et al. (6)	29.6%	47.1%
Yaprak Bayrak B et al. (7)	25%	27.6%
Pereira C (8).	26.3%	26.6%
Baraf L et al. (9)	38%	58%
Godoi Cavalheiro B et al. (10)	16%	17%
Azzam EZ et al. (12)	18.2%	33.3%

Importantly, however, our findings should be interpreted within the context of a selected surgical population and should not be extrapolated directly to all nodules classified as BCs III or IV in general endocrine practice. Although BC IV nodules were associated with larger malignant tumor size and microcarcinomas were less frequent in BC IV than in BC III, cytological category itself was not an independent predictor of malignancy in multivariable analysis. This suggests that within a surgical cohort, the distinction between BC III and BC IV alone may be insufficient for individual risk stratification and should be interpreted together with age, ultrasound findings, repeat cytology, clinical context, and patient preference.

Our findings also support the concept that Bethesda category alone should not be considered sufficient for contemporary thyroid nodule risk stratification. Although BCs III and IV are associated with an increased risk of malignancy compared with benign cytological categories, clinical decision-making requires integration of multiple parameters, including ultrasonographic characteristics, clinical risk factors, patient preferences, and, when available, molecular testing. In our cohort, younger age was independently associated with both malignancy (adjusted OR 1.74; 95% CI 1.23–2.47; p=0.002) and lymph node metastases (adjusted OR 4.56; 95% CI 1.19–17.53; p=0.03), whereas Bethesda category itself was not an independent predictor of malignancy after adjustment for other factors. These findings emphasize the importance of a comprehensive, individualized risk assessment rather than relying solely on cytological classification.

Among the variables analyzed, younger age emerged as the most consistent factor associated with malignancy. Younger patients had a significantly higher odds of malignancy in both univariable and multivariable analysis. However, this finding should be interpreted cautiously. Although age remained an independent predictor in the multivariable model, its performance as an isolated predictor was poor, with an ROC-derived AUC of only 0.55. Despite statistical significance, the multivariable model demonstrated modest diagnostic accuracy, with an area under the ROC curve (AUC) of 0.60, reflecting only slight improvement over chance. This, together with low pseudo R² values, indicates that age alone has limited discriminative value and was retained in the multivariable model because of its established clinical relevance rather than its standalone predictive performance. The ROC derived age cutoff of 50 years should therefore be regarded as a pragmatic threshold used for age categorization in the regression analyses rather than a clinically meaningful discriminator of malignancy. The model’s limited performance underscores the complexity of preoperative malignancy prediction in indeterminate thyroid nodules. In the subgroup of patients with TC, age <50 years was also associated with lymph node metastasis. Although this observation is clinically interesting, it should be considered exploratory because the number of metastatic events was limited, confidence intervals were wide, and the low events-per-variable ratio increased the risk of model overfitting. Therefore, these data are insufficient to support stronger conclusions regarding metastatic risk or to justify a more aggressive surgical strategy on the basis of age alone. Some studies report a higher risk in younger patients, with age ≤50 years identified as an independent predictor of malignancy (15), while others demonstrate an increased risk in older individuals (≥40 years) (12). Moreover, in several analyses, age was not an independent predictor after adjustment for clinical and ultrasonographic factors, suggesting that its role may be limited compared to imaging features and biochemical markers (16). Therefore, studies on larger patient cohorts are needed to better determine the impact of age on the risk of malignancy.

The interpretation of BC III nodules is additionally limited by the lack of information on the atypia of undetermined significance (AUS) and follicular lesion of undetermined significance (FLUS) subcategories. Previous studies have shown that cytological subtyping within BC III may substantially modify the risk of malignancy (17–21), and the absence of these data in our cohort may have influenced the observed rates. In the study by Johnson et al., the risk of malignancy for AUS (33.3%–26.0%) was higher than for FLUS (7.7%–5.0%) (18). Similarly, Gan et al. demonstrated a higher risk of malignancy for nuclear atypia (36.8%) compared to architectural atypia (14.7%) (19), which was also observed in the study by Terzi NK et al. (20). Although ultrasound features are known to improve preoperative risk stratification in indeterminate nodules (22), EU-TIRADS data in our study were incomplete and therefore not suitable for robust analysis. This represents an important limitation and highlights the need for more standardized preoperative assessment.

Numerous studies have also analyzed the ultrasound features of nodules classified as BCs III and IV (13, 14, 21, 22). Hypoechogenicity and the presence of microcalcifications have been identified as risk factors for malignancy in these groups (13). It has also been shown that irregular borders, solitary nodules, hypoechogenicity, microcalcifications, and a taller-than-wide shape increase the risk of malignancy in thyroid nodules (14, 22–24), as do larger nodule size (≥2 cm) and younger age (<65 years) (22).

CLT also requires careful interpretation. In our descriptive analyses, benign lesions predominated among nodules with coexisting thyroiditis, but CLT was not an independent predictor of malignancy in the regression model. Other studies have found no association between CLT and the risk of malignant tumors in BC III (25, 26). Taken together with the conflicting literature, this suggests that CLT should not currently be considered a reliable decision-making marker for surgical referral in patients with BC III or IV cytology.

The present study provides additional insight into the histopathological spectrum of BCs III and IV thyroid nodules by incorporating the category of LRTN introduced in the 2022 WHO Classification of Thyroid Tumors. In our cohort, benign lesions were the most frequent outcome in both BCs III and IV nodules (62.5% and 55.7%, respectively), followed by malignant tumors (30.4% and 32.8%) and LRTN (7.1% and 11.5%). These findings indicate that the traditional dichotomous classification of histopathological outcomes as benign or malignant may not fully reflect the biological spectrum of indeterminate thyroid nodules. Previous studies assessing BCs III and IV nodules have mainly relied on this binary classification; therefore, reported malignancy rates may not be directly comparable with contemporary data incorporating LRTN as a separate category. The identification of LRTN, including NIFTP, FT-UMP, and WDT-UMP, may have important clinical implications by supporting more individualized management of BCs III and IV nodules. These entities reduce the apparent burden of overt malignancy within indeterminate cytology and highlight the risk of overtreatment when all BCs III and IV nodules are considered potentially aggressive cancers. More accurate preoperative identification of low-risk lesions remains an important clinical challenge. The reclassification of borderline follicular-patterned lesions, particularly NIFTP, has further implications for the interpretation and management of indeterminate thyroid nodules. Previous studies have demonstrated that inclusion of NIFTP within low-risk categories reduces reported malignancy rates and indicates that some BCs III and IV nodules have minimal aggressive potential (27, 28). Similarly, FT-UMP and WDT-UMP may occur in both BCs III and IV, emphasizing that indeterminate cytology does not necessarily indicate malignant behavior. Żyłka et al. showed that FT-UMP constitute a significant proportion of low-risk thyroid lesions and may be classified cytologically as BCs III or IV, most commonly Bethesda IV. These findings support careful interpretation of indeterminate cytology and individualized management, including repeat FNAB and detailed ultrasound assessment before surgery (29).

In our cohort, total thyroidectomy was performed in more than 80% of patients in both cytological categories; however, this finding should not be interpreted as evidence that total thyroidectomy is optimal for all Bethesda III and IV nodules. The extent of surgery was likely influenced by factors not fully captured in our analysis, including bilateral nodular disease, referral patterns, surgeon judgment, patient preference, and limited access to molecular diagnostics. Therefore, our findings support an individualized approach to surgical decision-making based on the overall preoperative risk profile. In the study by Payne et al., the role of preoperative molecular testing in determining the extent of surgery was emphasized (30). Molecular diagnostics may improve management of BCs III and IV nodules by enabling more accurate risk stratification. According to the authors, high-risk mutations, such as BRAF and TERT, may support total thyroidectomy, whereas low-risk molecular profiles, including RAS-like alterations, may justify more conservative approaches such as hemithyroidectomy or active surveillance. However, the availability of molecular testing remains limited in the Polish healthcare system and many other European countries. Therefore, careful ultrasound assessment and repeat FNAB when indicated remain essential components of preoperative decision-making.

Given our findings indicating the presence of malignant tumors in these two categories, it is important to emphasize the importance of a thorough preoperative assessment in order to identify patients requiring surgery and to determine the extent of the procedure. It is recommended that a repeat FNAB be performed, reserving surgery for cases of persistent atypia, suspicious features on ultrasound, or other clinical indications (31). According to the 2025 ATA guidelines, in cases of thyroid nodules of indeterminate nature (Bethesda III and IV), lobectomy is generally considered sufficient for solitary nodules without high-risk features, measuring <4 cm, and with a negative or low-risk molecular profile. Total thyroidectomy may be considered when the nodule is ≥4 cm, shows suspicious features on ultrasound, or has positive or high-risk molecular results. It is also indicated in the presence of high-risk clinical factors, such as a history of radiation exposure, a strong family history of thyroid cancer, bilateral nodular disease, or the patient’s preference for definitive treatment (32).

Finally, the limitations of our study should be acknowledged. First, it was retrospective and based exclusively on surgically treated patients, which introduces selection bias and limits the generalizability of the observed malignancy rates. Second, ultrasound and FNAB were performed at multiple referring centers, reducing preoperative standardization. Third, EU-TIRADS classification was incomplete and could not be reliably incorporated into the main analysis. Fourth, BC III subcategories (AUS vs FLUS) were unavailable. Fifth, molecular testing was not routinely available. Finally, the exploratory analysis of lymph node metastasis should be interpreted with caution due to the limited number of events and incomplete nodal classification in some cases. Despite these limitations, our study provides clinically relevant data from a large contemporary surgical cohort of BC III and IV thyroid nodules classified according to the current WHO framework. The findings support a cautious and individualized interpretation of indeterminate cytology and underscore the need for better preoperative tools to distinguish benign, low-risk, and malignant lesions before surgery.

## Conclusions

Malignant lesions accounted for approximately one-third of BCs III and IV thyroid nodules, highlighting the need for careful preoperative risk assessment, including ultrasound evaluation and repeat FNAB when indicated. The identification of low-risk thyroid neoplasms as a separate category emphasizes the heterogeneity of indeterminate nodules and supports individualized management. Younger age (<50 years) was associated with a higher risk of malignancy; however, age alone should not determine surgical strategy.

## Data Availability

The raw data supporting the conclusions of this article will be made available by the authors, without undue reservation.
