# Supplementary material for: Prevalence and predictors of malignancy in Bethesda III and IV thyroid nodules undergoing surgery
Source: Front Endocrinol (Lausanne). 2026 Aug 17;17:1859531. doi: 10.3389/fendo.2026.1859531 (PMC13526674; doi:10.3389/fendo.2026.1859531)
Supplement: Supplementary file 1 [file DataSheet1.docx]

*Figure S1. Receiver operating characteristic (ROC) curve for age as a predictor of malignancy in thyroid nodules (among both III and IV Bethesda groups), with the optimal cut-off point at 50 years determined by the Youden index (AUC = 0.55, 95% CI 0.50–0.60; sensitivity 53%, specificity 63%).*
